# The Physical Mechanisms of *Drosophila* Gastrulation: Mesoderm and Endoderm Invagination

**DOI:** 10.1534/genetics.119.301292

**Published:** 2019-12-11

**Authors:** Adam C. Martin

**Affiliations:** Department of Biology, Massachusetts Institute of Technology, Cambridge, Massachusetts 02142

**Keywords:** adherens junction, apical constriction, cytoskeleton, FlyBook, GPCR, morphogen, morphogenesis, myosin, Rho

## Abstract

A critical juncture in early development is the partitioning of cells that will adopt different fates into three germ layers: the ectoderm, the mesoderm, and the endoderm. This step is achieved through the internalization of specified cells from the outermost surface layer, through a process called gastrulation. In *Drosophila*, gastrulation is achieved through cell shape changes (*i.e.*, apical constriction) that change tissue curvature and lead to the folding of a surface epithelium. Folding of embryonic tissue results in mesoderm and endoderm invagination, not as individual cells, but as collective tissue units. The tractability of *Drosophila* as a model system is best exemplified by how much we know about *Drosophila* gastrulation, from the signals that pattern the embryo to the molecular components that generate force, and how these components are organized to promote cell and tissue shape changes. For mesoderm invagination, graded signaling by the morphogen, Spätzle, sets up a gradient in transcriptional activity that leads to the expression of a secreted ligand (Folded gastrulation) and a transmembrane protein (T48). Together with the GPCR Mist, which is expressed in the mesoderm, and the GPCR Smog, which is expressed uniformly, these signals activate heterotrimeric G-protein and small Rho-family G-protein signaling to promote apical contractility and changes in cell and tissue shape. A notable feature of this signaling pathway is its intricate organization in both space and time. At the cellular level, signaling components and the cytoskeleton exhibit striking polarity, not only along the apical–basal cell axis, but also within the apical domain. Furthermore, gene expression controls a highly choreographed chain of events, the dynamics of which are critical for primordium invagination; it does not simply throw the cytoskeletal “on” switch. Finally, studies of *Drosophila* gastrulation have provided insight into how global tissue mechanics and movements are intertwined as multiple tissues simultaneously change shape. Overall, these studies have contributed to the view that cells respond to forces that propagate over great distances, demonstrating that cellular decisions, and, ultimately, tissue shape changes, proceed by integrating cues across an entire embryo.

EPITHELIA are abundant tissue types in metazoan organisms whose structure is established early in embryonic development (Honda 2017). Two defining properties of epithelia are that their constituent cells are (1) physically linked through adhesions to form a sheet, or layer of cells, with important barrier and compartmentalization functions; and (2) polarized across the sheet such that the protein composition on the outer/lumenal side (*i.e.*, apical) of the sheet differs from that on the inner side (*i.e.*, basal). Because epithelial structure is established early in embryonic development, epithelia have to undergo extensive shape changes in order to give rise to the final shape of organs and organisms (Kasza and Zallen 2011; Lecuit *et al.* 2011; Heisenberg and Bellaiche 2013; Heer and Martin 2017).

The formation of epithelial shape is a process termed epithelial morphogenesis. In *Drosophila*, and in other organisms, epithelial morphogenesis follows a stereotypical regulatory structure (Figure 1A). First, morphogens create a pattern of gene expression across the embryo. Second, this gene expression leads to the expression of signals, often extracellular, that promote cellular force generation and morphogenesis. This review will focus on actomyosin contractility as the mode of force generation, as opposed to actin polymerization-based protrusion, because of a preponderance of evidence suggesting that contractility is a major driver of epithelial sculpting (Quintin *et al.* 2008). Contractility can promote cell and tissue shape changes by driving the contraction or shrinkage of a cellular domain.

**Figure 1:**
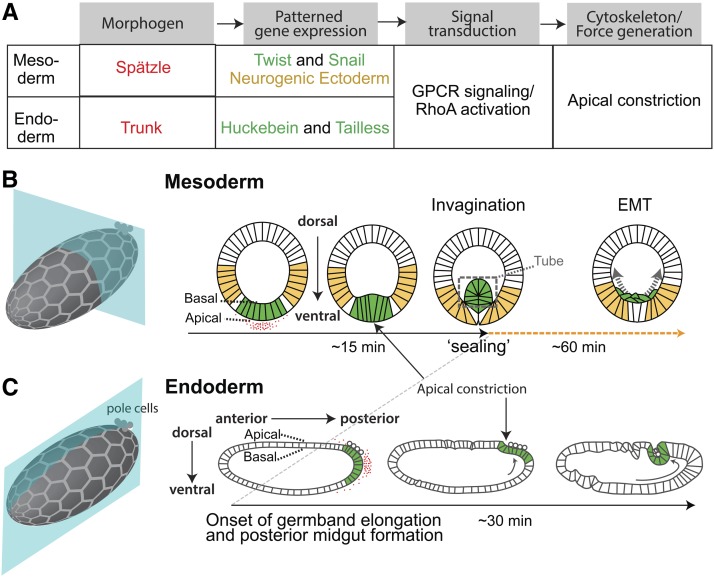
*Drosophila* gastrulation overview. (A) Flow chart showing the regulation of cell shape changes that accompany *Drosophila* gastrulation. Colored text matches the colors in (B and C). (B) Cartoon showing mesoderm invagination in *Drosophila*. Hexagonal mesh illustrates plane of epithelium, but cell size is not to scale. Red shows pattern of active Spätzle. Green illustrates presumptive mesoderm cells, which express Twist and Snail in response to high levels of Dorsal. Yellow cells show region of ectoderm, which is specified by low levels of Dorsal. (C) Cartoon showing posterior endoderm invagination. Hexagonal mesh illustrates plane of epithelium, but cell size is not to scale. Red shows distribution of the signaling ligand Trunk. Green illustrates presumptive endoderm cells expressing Huckebein and Tailless. Arrow shows the direction of germband elongation.

Cellular contractility is most commonly driven by two proteins, actin and nonmuscle myosin 2 (myosin 2), which are regulated through their assembly into filaments (Murrell *et al.* 2015). Individual globular actin (G-actin) subunits assemble into filamentous actin (F-actin); actin polymerization is regulated at the level of actin nucleation (by formins and the Arp2/3 complex) and elongation (promoted by Formins and Ena/VASP proteins, and antagonized by Capping protein) (Goode and Eck 2007; Campellone and Welch 2010; Edwards *et al.* 2014). Myosin 2 forms polymers called bipolar filaments, and is regulated by phosphorylation of the regulatory light chain of the molecule (Heissler and Sellers 2016). Myosin 2 bipolar filaments have the motor heads facing opposite directions, which allows motors on opposing sides of the bipolar filament to bind and walk along F-actin arrays, thereby sliding them past each other (Murrell *et al.* 2015).

Gastrulation in the *Drosophila* embryo has served as a major model system for understanding the connection between gene expression and epithelial morphogenesis (Figure 1A) (Leptin 2005). Gastrulation is the process by which a single-layered embryo is converted to multiple “germ” layers. Like many animals, *Drosophila* establishes domains of cells that invaginate to form either mesoderm or endoderm structures (Figure 1, B and C). *Drosophila* mesoderm formation involves the inward folding of an epithelial sheet, which results in cell invagination from the outer layer and subsequent formation of an inner layer (Leptin and Grunewald 1990; Sweeton *et al.* 1991) (Figure 1B). *Drosophila* gastrulation has played an important role in advancing our understanding of the mechanisms through which actomyosin contractility can shape an embryo (Martin and Goldstein 2014). While some of the details of embryo structure are specific to *Drosophila*, many of the molecular and cellular mechanisms regulating *Drosophila* gastrulation are conserved in different contexts. For example, at the cell level, invagination is promoted by a widely utilized cell shape change called apical constriction (Sawyer *et al.* 2010) (Figure 1, B and C). Apical constriction involves cells contracting on one side of the epithelial layer (*i.e.*, outer or apical), which changes cell shape from columnar to wedge-shaped, thus, promoting inward tissue curvature (Heer *et al.* 2017). At the molecular level, having a secreted ligand to stimulate apical actomyosin contractility through G-protein coupled receptor signaling is a theme shared by multiple cell types, such as endothelial cells (Shen *et al.* 2009). At the tissue level, assembly of multicellular actomyosin networks, which propagate force across the hundreds of cells, have been shown to be critical for its proper sculpting (Martin *et al.* 2010; Yevick *et al.* 2019). Similarly organized actomyosin networks and means of force propagation have also been shown to operate during gastrulation and neural tube closure in vertebrates (Pfister *et al.* 2016; Galea *et al.* 2017), thus rendering *Drosophila* an increasingly relevant model organism for uncovering the principles that underlie collective cell behavior and morphogenesis.

## Gene Regulation and Cell Specification in *Drosophila* Gastrulation

### Morphogen signaling and dorsal activation

During *Drosophila* gastrulation, presumptive mesoderm and endoderm cells invaginate sequentially (Figure 1, B and C). This chapter will focus on mesoderm invagination, for which the connection between gene expression patterns and morphogenesis is best understood. However, the logic that underlies mesoderm invagination is also true for posterior midgut invagination, and these connections and differences will be discussed.

**Figure 2:**
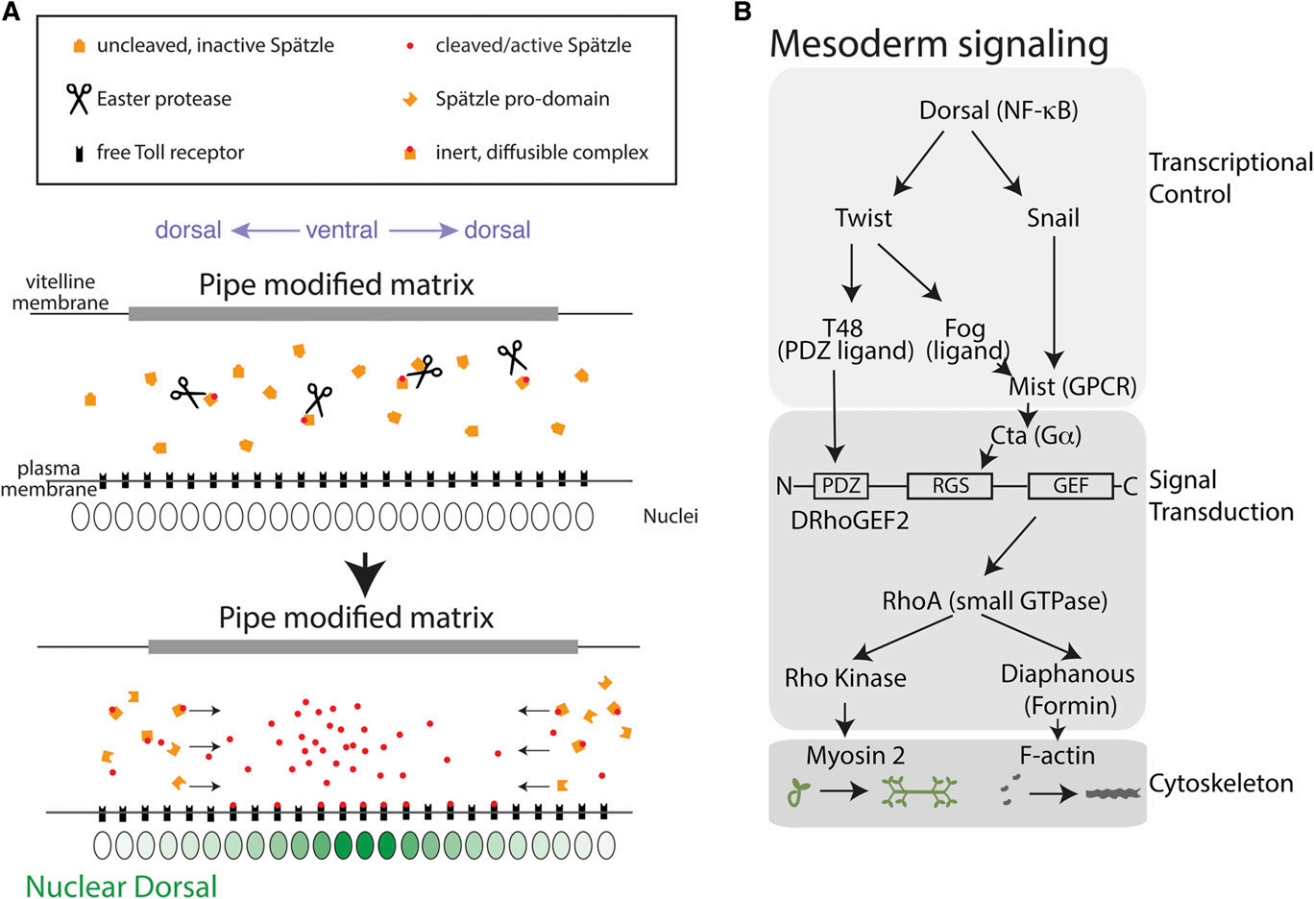
Signaling that promotes mesoderm invagination. (A) Pathway for Spätzle activation and Dorsal nuclear translocation. Spätzle is produced in an inactive proform and is cleaved by a serine protease (Easter) that is activated within the Pipe domain. Binding of cleaved Spätzle to Toll releases the inhibiting prodomain. Active Spätzle bound to its prodomain is free to diffuse. Prodomain is generated preferentially outside the Pipe domain leading to diffusive flux of Spätzle toward the ventral midline. (B) Genetic pathway for cytoskeletal activation in the *Drosophila* mesoderm. Domain organization of DRhoGEF2 protein is shown, see text for description.

*Drosophila* dorsal–ventral polarity is established in the mother’s ovary, where the reciprocal interaction between oocyte and surrounding follicle cells establishes the major body axes prior to fertilization (Roth 2003; Stein and Stevens 2014). Ultimately, signaling via the Toll pathway leads to the graded distribution of nuclear Dorsal in the embryo, which peaks at the ventral midline and decreases in the direction of the dorsal side of the embryo (Figure 2A) (Steward *et al.* 1988; Roth *et al.* 1989; Rushlow *et al.* 1989; Steward 1989). The morphogen that specifies dorsal–ventral polarity and forms a ventral–dorsal gradient is called Spätzle, which binds to the Toll receptor and promotes nuclear translocation of Dorsal (NF-κB) (Figure 2A). The Spätzle protein is synthesized in an inactive form that is proteolytically activated (Figure 2A). Proteolytic activation of Spätzle occurs preferentially in a region of the embryo that was in contact with ovarian follicle cells expressing the *pipe* gene (Moussian and Roth 2005; Stein and Stevens 2014). The *pipe* gene encodes a heparan sulfate 2-O-sulfotransferase that modifies the vitelline membrane or some other extracellular matrix component (Sen *et al.* 1998). It is within this domain of *pipe* expression that the penultimate protease in a protease cascade, Easter, is activated, which culminates in Spätzle activation by cleavage (Cho *et al.* 2012; Rahimi *et al.* 2019). Restriction of Easter-mediated Spätzle activation to a domain of a given width is ensured through negative regulation by a serine protease inhibitor, Serpin27A (Spn27A) (Chang and Morisato 2002; Hashimoto *et al.* 2003; Ligoxygakis *et al.* 2003).

Although *pipe* expression defines the limits of Spätzle activation, a more refined pattern of active Spätzle is established by a shuttling mechanism that involves active Spätzle rebinding its prodomain, forming a diffusible, but inert, complex (Haskel-Ittah *et al.* 2012). Because the free prodomain displays a higher concentration at the lateral regions of the embryo, prodomain cleavage and release of free active Spätzle will have different consequences at different regions. In the lateral region, where the concentration of the free prodomain is high, the released ligand will rebind a prodomain molecule and diffuse. However, when active Spätzle is released in the ventral region where the concentration of the prodomain is low, it has a higher chance of binding the Toll receptor. More free Spätzle molecules will be released in the ventral region, forming a dynamic and robust morphogen gradient (Shilo *et al.* 2013). Binding to Toll functions as a “sink,” and, hence, the diffusive flux of active Spätzle-prodomain complexes toward the ventral midline results in an active Spätzle distribution that narrows over time (Rahimi *et al.* 2019), resulting in the graded nuclear translocation of the transcription factor, Dorsal, within the *pipe* domain.

Dorsal’s various target genes have different thresholds for activation: high concentrations of nuclear Dorsal lead to the expression of mesoderm-specific genes (*i.e.*, *twist* and *snail*, which have highest threshold for expression), whereas lower nuclear Dorsal concentrations lead to the expression of different genes in the neurogenic ectoderm (Chopra and Levine 2009) (Figure 1B, yellow). In this manner, a single morphogen gradient establishes gene expression domains at different positions along the dorsal–ventral axis, including mesoderm specification in the most ventral domain. High nuclear Dorsal promotes the expression of two transcription factors, Twist and Snail, in the ventral-most cells of the presumptive mesoderm (Figure 2B). The combination of Dorsal, Twist, and Snail can, in turn, induce or repress the expression of other genes that promote contractility and cell shape changes, which result in mesoderm invagination (Leptin 1991).

### Gene expression: twist

Twist is a transcriptional activator that induces the expression of hundreds of genes (Furlong *et al.* 2001). After loss of Twist activity, limited cell shape changes still occur, but the large-scale movement of the mesoderm tissue is lost or unsustained during later stages of development (Leptin and Grunewald 1990; Seher *et al.* 2007). Analysis of myosin 2 dynamics in *twist* mutants demonstrates that apical contractions are transient and reversible, resulting in a failure to sustain cell shape changes (*i.e.*, apical constriction) (Martin *et al.* 2009). Thus, Twist promotes sustained apical contractility, which is required for myosin 2 to form a supracellular meshwork across the apical surface (Martin *et al.* 2010).

Two to three of the genes induced by Twist act in concert to promote apical contractility and mesoderm invagination during gastrulation (Seher *et al.* 2007). First, Twist promotes the activation of a G protein-coupled receptor (GPCR) pathway by activating the expression of *folded gastrulation* (*fog*), the signaling ligand for this pathway (Costa *et al.* 1994) (Figure 2B). Most of the downstream components of this GPCR pathway are maternally supplied, including heterotrimeric G protein subunits, the small GTPase RhoA and its regulators, and cytoskeletal proteins. For example, the gene encoding the heterotrimeric Gα_12/13_ protein associated with this pathway, *concertina* (*cta*), has a maternal effect phenotype similar to zygotic *fog* mutants (Schupbach and Wieschaus 1989; Parks and Wieschaus 1991). Mutations in most of the components of this GPCR pathway do not prevent mesoderm invagination, but result in uncoordinated apical constriction, where some cells constrict while others exhibit delayed or abnormal constriction (Sweeton *et al.* 1991; Manning *et al.* 2013; Xie *et al.* 2016).

Several GPCRs have been identified as Fog receptors. One GPCR, called Mesoderm-invagination signal transducer (*mist*, also called Methuselah-like 1), is specifically expressed in the mesoderm in a manner that requires Snail activity (Manning *et al.* 2013) (Figure 2B). In contrast, another GPCR, called Smog, is ubiquitously present in the early embryo (Kerridge *et al.* 2016). Differential GPCR endocytosis between mesoderm and ectoderm cells is another way through which differential contractility is achieved between these cell populations (Jha *et al.* 2018). The G protein receptor kinase (Gprk2) and a β-arrestin (Kurtz) are maternally supplied, and modulate GPCR signaling in the mesoderm and ectoderm (Fuse *et al.* 2013; Jha *et al.* 2018; Chai *et al.* 2019). Kurtz also modulates Toll signaling (Anjum *et al.* 2013), suggesting that signal termination plays a critical role in defining the region of the presumptive mesoderm, as well as differences between mesoderm and ectoderm. Although differences in the modulation of GPCR signaling could contribute to contractility differences between mesoderm and ectoderm, the fact that ectopic Fog expression results in relatively uniform apical myosin 2 activity along the dorsal–ventral axis strongly suggests that Fog expression is a main determinant that differentiates mesoderm behavior from that of the ectoderm (Morize *et al.* 1998; Dawes-Hoang *et al.* 2005). Importantly, the higher *fog*-dependent Smog homo-cluster formation and recruitment to plasma membrane invaginations in the mesoderm, indicate that Fog binds Smog in the mesoderm (Jha *et al.* 2018).

A second functional output of Twist expression is the expression of a transmembrane protein known as T48 (Kolsch *et al.* 2007). T48 has a cytoplasmic tail with a PSD95/Dlg1/ZO-1 (PDZ) domain interaction motif. Mutation of *T48*, on its own, has little consequence on mesoderm invagination. However, disruption of both *T48* and *cta* results in embryos that resemble *twist* mutants, suggesting that *T48* and GPCR signaling function in parallel to promote mesoderm invagination (Kolsch *et al.* 2007) (Figure 2B). In terms of Twist’s function in myosin 2 regulation, it is also the case that codepletion of both Fog and T48 results in a failure to sustain apical myosin 2 levels, which is a phenotype similar to that of Twist depletion (Martin *et al.* 2010).

Twist has several other targets that are also important for invagination. First, Twist cooperates with Dorsal to enhance *snail* expression. Twist-mediated *snail* expression appears to expand the *snail* domain, and to sustain high uniform *snail* levels in the presumptive mesoderm (Leptin 1991; Ip *et al.* 1992). Other Twist targets are the *tribbles* and *frühstart* genes, which play a permissive role in invagination by repressing cell divisions that, otherwise, disrupt the invagination process (Grosshans and Wieschaus 2000; Mata *et al.* 2000; Seher and Leptin 2000; Grosshans *et al.* 2003). The tumor necrosis factor (TNF) receptor-associated factor 4 (*traf4*) gene, which is required for the fine-tuning of apical adherens junction assembly, is another Twist target (Mathew *et al.* 2011).

### Gene expression: snail

In *Drosophila*, Snail can repress or activate gene expression (Rembold *et al.* 2014). Only a few Snail target genes have been identified to have functional importance during mesoderm invagination, but these targets provide insight into how contractility is patterned across the embryo. One family of genes that is repressed by Snail is the *Bearded* family of genes, which inhibit Neuralized-mediated endocytosis of the signaling ligand, Delta (Bardin and Schweisguth 2006; De Renzis *et al.* 2006). The *Bearded* family of genes encodes a set of proteins that inhibit the E3 ubiquitin ligase Neuralized (Lai *et al.* 2001; Yeh *et al.* 2001). The *neuralized* gene is expressed in the ventral mesoderm by Twist, and promotes apical constriction through an unknown mechanism (Perez-Mockus *et al.* 2017). The Bearded proteins inhibit Neuralized, but fail to do so in the ventral mesoderm because they are transcriptionally repressed by Snail (De Renzis *et al.* 2006). The derepression of *neuralized* in the ectoderm that occurs in *Bearded* mutants results in elevated contractility and junction remodeling in ectoderm cells, which causes the mesoderm to unfold after having initiated invagination (Chanet and Schweisguth 2012; Perez-Mockus *et al.* 2017). Inhibiting *neuralized* either through mutation or ectopic expression of *Bearded* genes in the mesoderm does not completely recapitulate the phenotype of a *snail* mutant, which suggests that other Snail targets are critical for mesoderm invagination (Perez-Mockus *et al.* 2017).

Another gene that is repressed by Snail is *wntD*. Counterintuitively, *wntD* expression is induced in the presumptive mesoderm by Twist, but is repressed by Snail, resulting in low *wntD* expression (Ganguly *et al.* 2005). The *wntD* gene functions as a feedback inhibitor of the Toll/Dorsal pathway, with ectopic *wntD* expression blocking Dorsal activation (Ganguly *et al.* 2005; Gordon *et al.* 2005). The presence of feedback inhibition in this genetic network appears to promote robustness in the positional expression of genes downstream of Dorsal (Rahimi *et al.* 2016, 2019).

In contrast to gene repression, Snail activates the expression of the GPCR *mist* in the ventral mesoderm (Figure 2B) (Manning *et al.* 2013). *mist* expression in the mesoderm depends on Snail (Figure 2B). However, *snail* mutants result in a more severe reduction of actomyosin contractility than *twist* mutants, failing to exhibit even pulsatile myosin 2 dynamics (Martin *et al.* 2009). Furthermore, in contrast to *snail* mutants, *mist* mutants still undergo apical myosin 2 activation and mesoderm invagination (Manning *et al.* 2013; Kerridge *et al.* 2016), suggesting that *mist* is not Snail’s only functional target. Whether *bearded* gene repression and activating *mist* expression together account for all of Snail’s function in mesoderm invagination has not yet been tested.

### Posterior midgut invagination

Like mesoderm invagination, endoderm invagination also involves patterned gene expression leading to the induction of a signaling cascade that promotes cell and tissue shape changes (Figure 1, A and C). In the case of the posterior endoderm or posterior midgut, *fog* expression is induced by terminal transcription factors *huckebein* and *tailless* (Costa *et al.* 1994). In contrast to mesoderm invagination, where *fog* mutants do not prevent invagination in spite of uncoordinated apical constriction, *fog* mutants completely inhibit posterior midgut invagination (Costa *et al.* 1994). Thus, *fog* is required for posterior midgut invagination, but not for mesoderm invagination. It is not clear why this is the case, but one possibility is that mechanical coupling either between cells themselves, or between cells and the overlying extracellular matrix, plays a bigger role during endoderm invagination than during mesoderm invagination. For example, posterior midgut invagination involves an intercellular mechanical signaling relay, which could depend on Fog signaling (Pouille *et al.* 2009; Mitrossilis *et al.* 2017; Bailles *et al.* 2019). Another difference is that part of the posterior midgut tissue is attached to the overlying vitelline membrane via integrin-mediated adhesion, which plays a role in shaping this invagination (Bailles *et al.* 2019; Münster *et al.* 2019).

## RhoA Signaling—the Importance of Dynamics

Downstream of the GPCR signaling pathway induced by *snail* and *twist* transcription is DRhoGEF2, a guanine nucleotide exchange factor (GEF) for the small GTPase RhoA (Barrett *et al.* 1997; Hacker and Perrimon 1998). RhoA signaling activates actomyosin contractility by coordinately activating myosin 2 and F-actin assembly (Figure 2B) (Etienne-Manneville and Hall 2002; Jaffe and Hall 2005). DRhoGEF2 is maternally deposited into the embryo, and the maternal contribution is required for mesoderm invagination (Barrett *et al.* 1997; Hacker and Perrimon 1998). DRhoGEF2 is a member of a family of RhoGEFs, including Leukemia-associated Rho GEF (LARG) and PDZ-GEF in humans, which function downstream of heterotrimeric G proteins and also contain PDZ domains (Fukuhara *et al.* 2000). DRhoGEF2 has an N-terminal PDZ domain that could interact with the PDZ-binding motif of T48 (Kolsch *et al.* 2007). In addition, DRhoGEF2 has a Regulator of G-protein signaling (RGS) domain, which is thought to interact with the Gα, Concertina. Finally, DRhoGEF2 has a C-terminal DH-PH domain that is thought to be the catalytic GEF domain that activates RhoA. DRhoGEF2 responds to parallel inputs from Concertina and T48 to stimulate myosin 2 contractility (Figure 2B) (Kolsch *et al.* 2007).

**Figure 3:**
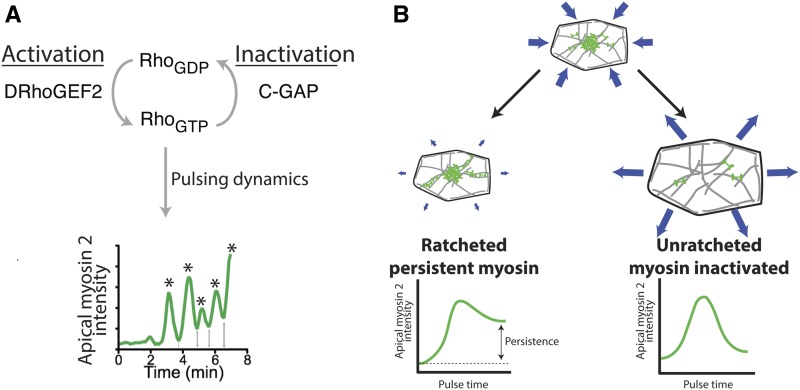
The dynamics of RhoA regulation in mesoderm cells. (A) Cyclical activation and inactivation of RhoA. DRhoGEF2 promotes formation of active RhoA-GTP. RhoA is inactivated by C-GAP. The combination of DRhoGEF2 and C-GAP is required for pulses of myosin 2 activation (asterisks). (B) RhoA activity levels determine the outcome of contractile pulse. High RhoA activity levels maintain apical myosin 2 after pulse, which stabilizes cell shape and promotes ratcheted constriction. Low RhoA activity levels fail to maintain myosin 2 after the pulse, which results in cell shape relaxation and unratcheted constriction.

During apical constriction, RhoA is not simply turned on. Instead, downstream outputs of the RhoA pathway, such as apical Rho-Kinase recruitment and myosin 2 activation are dynamic (Martin *et al.* 2009; Vasquez *et al.* 2014), which require dynamic regulation of RhoA (Figure 3A) (Mason *et al.* 2016). Myosin 2 activity exhibits pulsing behavior with bursts of myosin 2 activation followed by either myosin 2 inactivation or remodeling (Figure 3B) (Martin *et al.* 2009). Pulsatile dynamics are also observed with a RhoA activity biosensor or by imaging DRhoGEF2 itself (Munjal *et al.* 2015; Mason *et al.* 2016).

Rho GTPase signaling turnover is a key feature of contractile systems, including apical constriction (Denk-Lobnig and Martin 2019). Apical constriction itself is dynamic, occurring as a series of pulses where phases of apex contraction are interrupted by phases of apex relaxation or stabilization (Martin *et al.* 2009). Contractile pulses are correlated with bursts of myosin 2 assembly in the middle of the apical surface (Figure 3B) (Martin *et al.* 2009; Blanchard *et al.* 2010; Rauzi *et al.* 2010). These contractile pulses are initiated by bursts of DRhoGEF2, which precede apical myosin 2 by ∼10 sec (Mason *et al.* 2016). Interestingly, a Rab protein, Rab35, precedes myosin 2 localization by ∼45–60 sec, further suggesting that membrane trafficking organizes signaling events upstream of DRhoGEF2 (Miao *et al.* 2019). In mesoderm cells, a RhoA GTPase activating protein (GAP) called Cumberland-GAP, C-GAP, or RhoGAP71E mediates pulse termination. C-GAP depletion results in a continuous increase in myosin 2 activation without periods of myosin 2 inactivation (Mason *et al.* 2016). The fact that removing C-GAP disrupts pulsing suggests that pulsing in mesoderm cells involves RhoA signaling dynamics (Figure 3A). Indeed, RhoA signaling has been observed to exhibit hallmarks of excitable dynamics in other systems, such as pulsatile and wave-like behavior (Bement *et al.* 2015; Bischof *et al.* 2017; Michaux *et al.* 2018; Segal *et al.* 2018).

**Figure 4:**
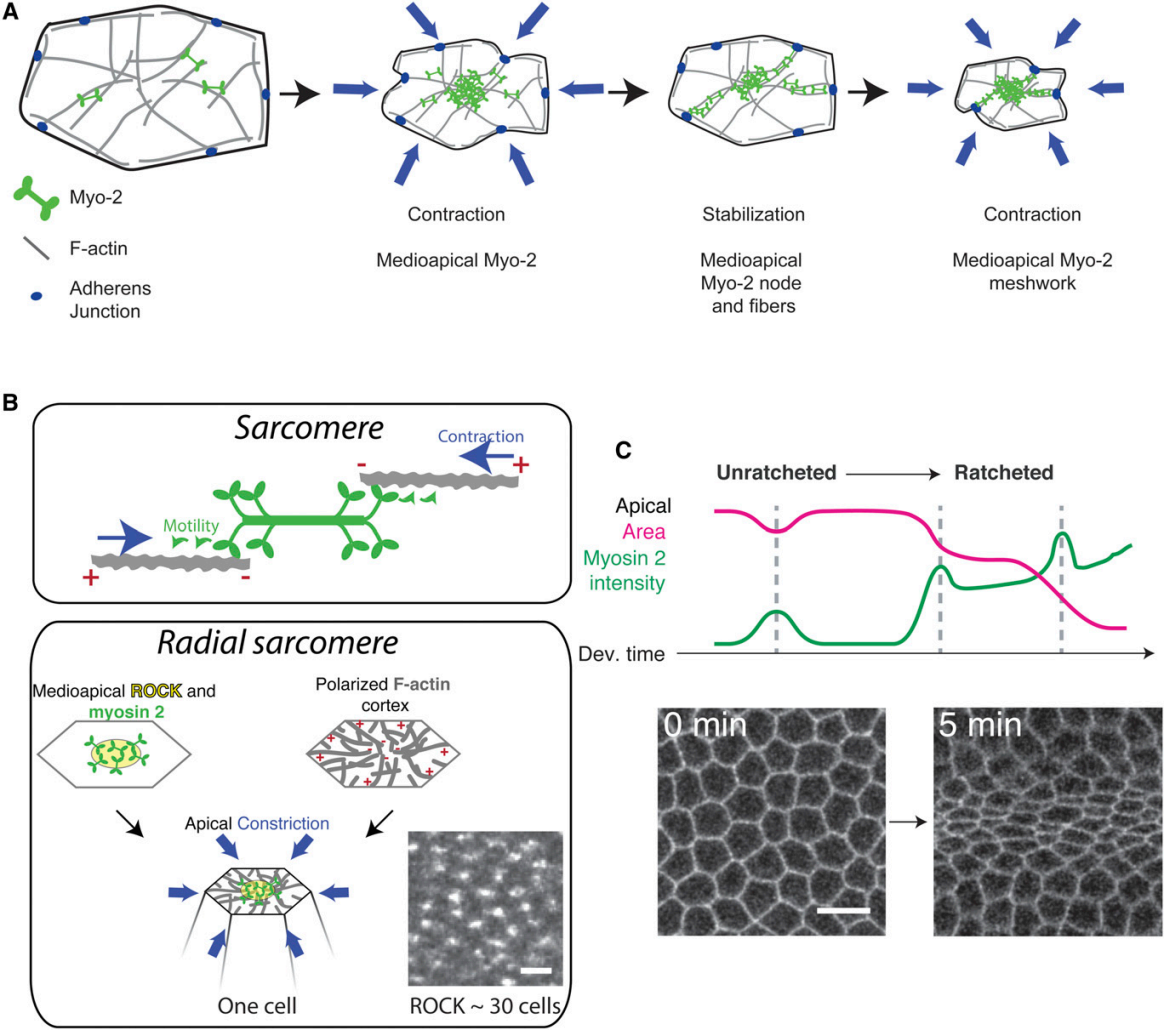
Spatial and temporal organization of myosin 2 contractility in mesoderm cells. (A) Ratcheted apical constriction of mesoderm cells. Medioapical actomyosin pulls centripetally on adherens junctions in stepwise constriction of the cell apex. (B) Apical cortex has a radial organization. Top, myosin 2 interacting with antiparallel actin filaments with plus ends facing out enables filament sliding and contraction. Bottom, medioapical myosin 2 activation and radial organization of actin filaments with outward facing plus ends enable actin network to be pulled toward center, constricting the apex. Image shows ROCK localization during apical constriction of the mesoderm, which shows a clear periodic pattern across the tissue—each spot represents the center of a contractile unit, which is a single cell. Bar, 3 μm. (C) Temporal progression of myosin 2 pulsing. Initially unratcheted pulses occur and then cells transition to having ratcheted pulses, which leads to sustained changes in apical area. Images show unconstricted and then constricted cell apices in the process of invagination. Images are reproduced from Chanet *et al.* (2017). Bar, 10 μm.

C-GAP and presumably control of RhoA activity level also determine the outcome of a contractile pulse. One outcome of a myosin 2 pulse is that cell apex constriction is stabilized (Figure 3B, left), such that the cell undergoes stepwise constriction like a ratchet (Figure 4A) (Martin *et al.* 2009). In *twist* mutants, apex constrictions resulting from myosin 2 pulses are predominantly reversed, with the apical area relaxing (Figure 3B, right). Whether apex constriction is stabilized or reversed is correlated with the amount of apical myosin 2 that persists following a pulse (Figure 3B) (Xie and Martin 2015). C-GAP overexpression disrupts apical myosin 2 persistence, which leads to cell apex relaxation and ineffective apical constriction (Mason *et al.* 2016). Interestingly, C-GAP overexpression is the perturbation that most closely resembles Twist depletion, suggesting that signaling downstream of Twist establishes a proper balance between RhoA activation by a GEF and inactivation by a GAP, which is critical for proper morphogenesis.

## Cytoskeletal Regulation and Organization During Invagination

RhoA activates myosin 2 through its effector Rho-Kinase (ROCK), which promotes myosin 2 activation by direct phosphorylation of myosin regulatory light chain and also by phosphorylating and inhibiting the myosin phosphatase (Amano *et al.* 1996; Mizuno *et al.* 1999; Winter *et al.* 2001). As with mammalian myosin 2, phosphorylation of the regulatory light chain (*spaghetti* *squash*, or *sqh*, in *Drosophila*) switches the motor activity from an “off” to an “on” state (Jordan and Karess 1997; Vasquez *et al.* 2016). During *Drosophila* gastrulation, ROCK is required for apical myosin 2 accumulation in apically constricting cells (Dawes-Hoang *et al.* 2005). Furthermore, acute ROCK inhibition results in the rapid (∼10 sec) disappearance of apical myosin 2, suggesting that ROCK is continuously required to balance inactivation by myosin phosphatase to maintain apical myosin 2 (Coravos and Martin 2016). Myosin phosphatase colocalizes with apical myosin 2 and promotes rapid myosin 2 turnover, which is required for myosin 2 pulsing (Vasquez *et al.* 2014; Munjal *et al.* 2015).

In addition to regulating myosin 2, RhoA also regulates actin cytoskeleton organization in the *Drosophila* mesoderm (Figure 2B) (Fox and Peifer 2007). Another RhoA effector, the formin Diaphanous (Goode and Eck 2007), is required for mesoderm invagination (Homem and Peifer 2008; Mason *et al.* 2013). However, despite RhoA activation in constricting cells, overall levels of cortical F-actin are decreased in mesoderm cells prior to furrow formation (Jodoin *et al.* 2015). This decline in cortical F-actin levels in mesoderm cells is attributed to higher levels of active Cofilin, an F-actin severing protein (Jodoin *et al.* 2015). Actin turnover, including formin-mediated F-actin assembly and cofilin-mediated disassembly, is important for maintaining intercellular connections in the tissue, as will be discussed in the next section.

### Spatial organization

Myosin 2 activation and F-actin assembly are spatially choreographed across the apical cortex of presumptive mesoderm cells. Active, GTP-bound RhoA and ROCK are enriched in the middle of the apical surface (medioapical), which promotes medioapical myosin 2 accumulation (Mason *et al.* 2013, 2016; Munjal *et al.* 2015) (Figure 4B). This polarization requires C-GAP, suggesting that precise regulation of RhoA activity establishes medioapical myosin 2 enrichment (Mason *et al.* 2016). In contrast, actin subunit incorporation at F-actin plus ends occurs predominantly at intercellular junctions (Coravos and Martin 2016). However, F-actin minus ends are medioapical and colocalize with myosin 2. The enrichment of the different F-actin ends in distinct apical regions suggests that the apical actin cortex is radially polarized (Figure 4B). Given that F-actin plus ends face outward and the minus ends and myosin 2 activation is predominantly medioapical, the apical actin cortex resembles a contractile unit, such as a sarcomere. Indeed, ROCK localization exhibits a polka-dot pattern across the tissue, with each dot representing the medioapical domain of one cell (Figure 4B).

Recent data has suggested that other cells may have a similar contractile organization. For example, the leading edge of the epidermis during *Drosophila* dorsal closure has a repeated pattern of myosin 2 localization that resembles “bars-on-a-string,” with each bar representing a single leading edge cell (Franke *et al.* 2005). Recent super-resolution microscopy of leading edge cells has shown that Ena—a protein that promotes F-actin plus end elongation—localizes to the adherens junctions, suggesting that F-actin plus ends are enriched facing outwards from a central myosin 2 bar (Manning *et al.* 2019). Given that leading edge cells have centrally localized myosin 2 and peripherally enriched F-actin plus ends, this topology again resembles a sarcomere. Adherens junctions exhibit F-actin plus end enrichment in several epithelial cell types (Tang and Brieher 2012; Verma *et al.* 2012); thus, this apical cortex organization may play a general role in nonmuscle contractility.

In mesoderm cells, the polarity of ROCK and myosin 2 is important for apical surface contraction, consistent with a sarcomere-like contraction (Mason *et al.* 2013; Coravos and Martin 2016). Furthermore, the rate of apical constriction is proportional to the ATPase activity of the myosin 2 motor (Vasquez *et al.* 2016), which is what is expected from a sarcomere-like model of contraction (Barany 1967). However, there are several important differences between the observed organization in mesoderm cells and that of a sarcomere. First, in mesoderm cells, the apical actin network is arranged radially around a signaling center, rather than being linear. Second, medioapical actomyosin networks are extremely dynamic and undergo self-organizing behaviors. One way in which actomyosin networks can undergo self-organization is through contraction-driven advection of plasma membrane associated proteins (Munro *et al.* 2004; Munjal *et al.* 2015), some of which (*i.e.*, Rho/ROCK advection) could feed back to regulate myosin 2 activity. Furthermore, as apical myosin 2 accumulates, medioapical actomyosin changes from medioapical spots to a fibrous organization that aligns relative to the mechanics of the surrounding tissue (Chanet *et al.* 2017). Thus, these apical actomyosin networks can exhibit self-organizing properties that enable them to change over time.

In addition to the radial organization of the apical F-actin cortex, the microtubule cytoskeleton also exhibits a radial polarity across the apex of constricting cells. During gastrulation, apical actomyosin contraction drives the formation of an medioapical microtubule organizing center, which promotes microtubule growth from the apical center out toward the cell junctions (Ko *et al.* 2019). Although the microtubule-associated protein EB1 binds DRhoGEF2 (Rogers *et al.* 2004), microtubule organization is not required for myosin 2 activation or initiating apical constriction, but promotes rapid actin dynamics in the apical cortex (Ko *et al.* 2019).

### Temporal organization

Cytoskeletal behavior during gastrulation is also organized in time. In the mesoderm, myosin 2 assembly occurs in pulses with phases of assembly followed by disassembly or remodeling (Martin *et al.* 2009). The outcome of a constriction is correlated with myosin 2 behavior after the pulse (Xie and Martin 2015). If medioapical myosin 2 is disassembled, then the apical cortex relaxes, and the cell fails to undergo net constriction (unratcheted pulse, Figure 3B). In contrast, if medioapical myosin 2 persists, then the constricted apical shape can be stabilized—an event that is often associated with formation of F-actin and myosin 2-containing fibers or cables that span the apical surface (ratcheted pulse, Figure 3B and Figure 4A). During mesoderm invagination, there is a temporal progression in cell behavior: initial pulses fail to exhibit myosin 2 persistence, and cells relax (Figure 4C, unratcheted); subsequent pulses lead to persistent myosin 2 accumulation, resulting in ratcheted contraction, which ultimately promotes collective tissue contraction (Figure 4C) (Xie and Martin 2015). Membrane trafficking through the Rab35 GTPase and its GEF, Sbf, is also important for ratcheted apical constriction (Miao *et al.* 2019). One effect of Rab35- and Sbf-depletion in embryos is that cells have heterogeneous myosin 2 levels, with some cells lacking and others having an abundance of myosin 2 (Miao *et al.* 2019). Because this phenotype is reminiscient of mutants in the Fog pathway (Parks and Wieschaus 1991; Sweeton *et al.* 1991; Costa *et al.* 1994; Xie *et al.* 2016), one hypothesis is that Sbf/Rab35 regulates GPCR signaling at plasma membrane invaginations (Jha *et al.* 2018). Alternatively, the removal of apical plasma membrane could be a process that acts in concert with contractility to change cell shape, as was proposed for *Xenopus* gastrulation (Lee and Harland 2010).

Work in *Caenorhabditis elegans* suggested that apical constriction is initiated by the onset of a molecular clutch that engages between the apical actomyosin cortex and the intercellular junctions (Roh-Johnson *et al.* 2012). It was suggested that a clutch mechanism was also responsible for the onset of mesoderm invagination in *Drosophila*, but several factors make this unclear. First, in the *Drosophila* mesoderm, pulsed actomyosin contractions are initially weak, and then strengthen over developmental time, making it difficult to know whether the initial contractions are not attached to intercellular junctions, or are simply too weak to change cell shape (Xie and Martin 2015). Second, adherens junctions are not initially present at the apical surface, but both adherens junctions and polarity proteins are pulled apically by actomyosin contractility (Weng and Wieschaus 2016, 2017). Therefore, initial contractions might not change cell shape, but still be connected to adherens junctions and be involved in pulling them apically. Finally, actomyosin contractility itself promotes the apical accumulation of adherens junction proteins during both *Drosophila* and *C. elegans* gastrulation (Marston *et al.* 2016; Weng and Wieschaus 2016). Therefore, greater coupling between the cell surface and the plasma membrane could result from an increase in the number of adhesion molecules recruited to adherens junction structures rather than a regulated link between the two. In summary, while a clutch-like mechanism was convincingly shown to trigger *C. elegans* gastrulation (Roh-Johnson *et al.* 2012), the trigger for *Drosophila* gastrulation appears to be more complicated, and to involve the coordination and change in both contractility and adherens junctions.

**Figure 5:**
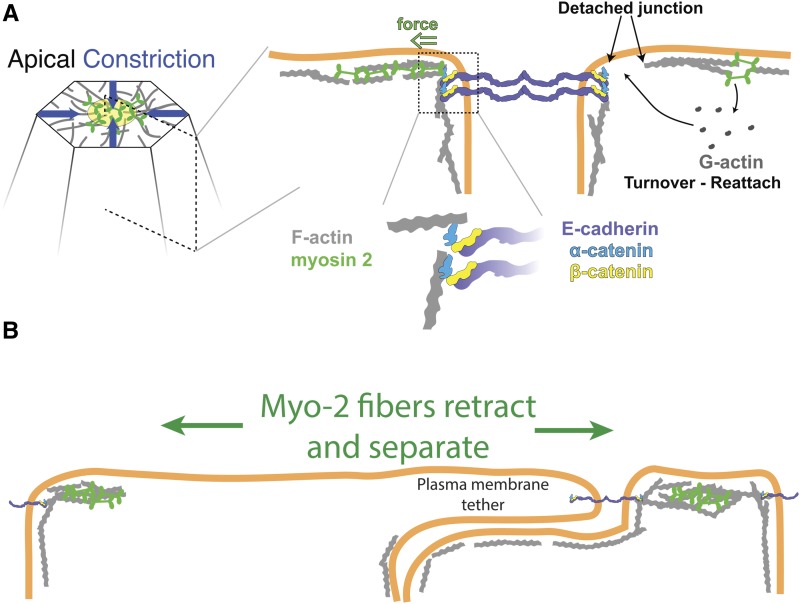
Integrating forces between cells during gastrulation. (A) Cartoon showing apical–basal cross-section through a wild-type cell, highlighting the adherens junction and its connection to the underlying F-actin cortex. The cell on the right illustrates a detachment event where actin turnover is required to reconnect the junction and medioapical actomyosin cortex. (B) Cartoon illustrating the outcome of depleting adherens junction components in the cell (*e.g.*, *armadillo* mutant). Note that medioapical actomyosin networks contract away from each other, with actomyosin fibers retracting. Also, note the plasma membrane tether that is pulled from the left-hand cell toward the cell on the right.

The F-actin cortex is also highly dynamic during apical constriction, and its dynamics are critical for force transmission across the tissue. During contractility the apical F-actin network continuously fragments (Jodoin *et al.* 2015). F-actin network fragmentation is especially prevalent next to adherens junctions, which separates the adherens junction from medioapical actomyosin (Figure 5A). Apical F-actin network turnover is responsible for repairing network fragmentation, thus, re-establishing the connection between the medioapical actomyosin and the adherens junctions (Figure 5A) (Jodoin *et al.* 2015). Importantly, the proper microtubule cytoskeleton organization is required to rapidly reestablish these lost connections, suggesting that cooperation between actin and microtubule cytoskeletal systems is critical for propagating force between cells (Ko *et al.* 2019).

While apical constriction in the endoderm cells of the posterior midgut is similar to that of the mesoderm (*e.g.*, apical constriction is pulsatile (Chanet *et al.* 2017), and myosin 2 is enriched medioapically), the distribution of medioapical myosin 2 is different. In contrast to mesoderm cells, where myosin 2 is organized into a medioapical node with fibers that span the apical surface (Figure 4A), endoderm cells contain myosin 2 organized into medioapical rings (Chanet *et al.* 2017). The cause of this different distribution will be discussed in the next section.

## Force Integration Across the Tissue and Resulting Cell Shape

For cells to promote a tissue-wide change, cellular forces have to be integrated across the tissue. During mesoderm invagination, adherens junctions are required to mechanically couple cells (Dawes-Hoang *et al.* 2005; Sawyer *et al.* 2009; Martin *et al.* 2010). Adherens junctions contain the adhesion receptor E-cadherin, which forms clusters that physically link cells on the extracellular side of the plasma membrane (Figure 5A) (Yap *et al.* 2015). On the cytoplasmic side of the adherens junctions, adaptor proteins such as α- and β- catenin and Canoe/Afadin link E-cadherin receptors to the underlying actomyosin cytoskeleton (Lecuit and Yap 2015; Vasquez and Martin 2016). All of these proteins are required to integrate forces across the invaginating mesoderm.

In *Drosophila*, adherens junctions initially form plaque-like structures known as spot adherens junctions before forming a continuous zonula adherens (Tepass and Hartenstein 1994). In mesoderm cells, fibrous actomyosin structures spanning the apical surface connect to spot adherens junctions in an end-on manner, which pulls adherens junctions centripetally, toward the center of the cell apex (Martin *et al.* 2009) (Figure 4A and Figure 5A). The weakest point of this transmission appears to be the connection between the actomyosin cytoskeleton and the cytoplasmic interface of the adherens junction. For example, live imaging of the F-actin cortex in wild-type embryos has revealed repeating fragmentation events that separate medioapical actomyosin from the adherens junctions (Figure 5A) (Jodoin *et al.* 2015). Furthermore, lowering adherens junction protein levels results in actomyosin networks tearing away from one side of an intercellular junction, which results in a plasma membrane tube or “tether” being pulled from the unattached cell toward the cell whose junctional attachment remains (Figure 5B) (Sawyer *et al.* 2009; Martin *et al.* 2010).

**Figure 6:**
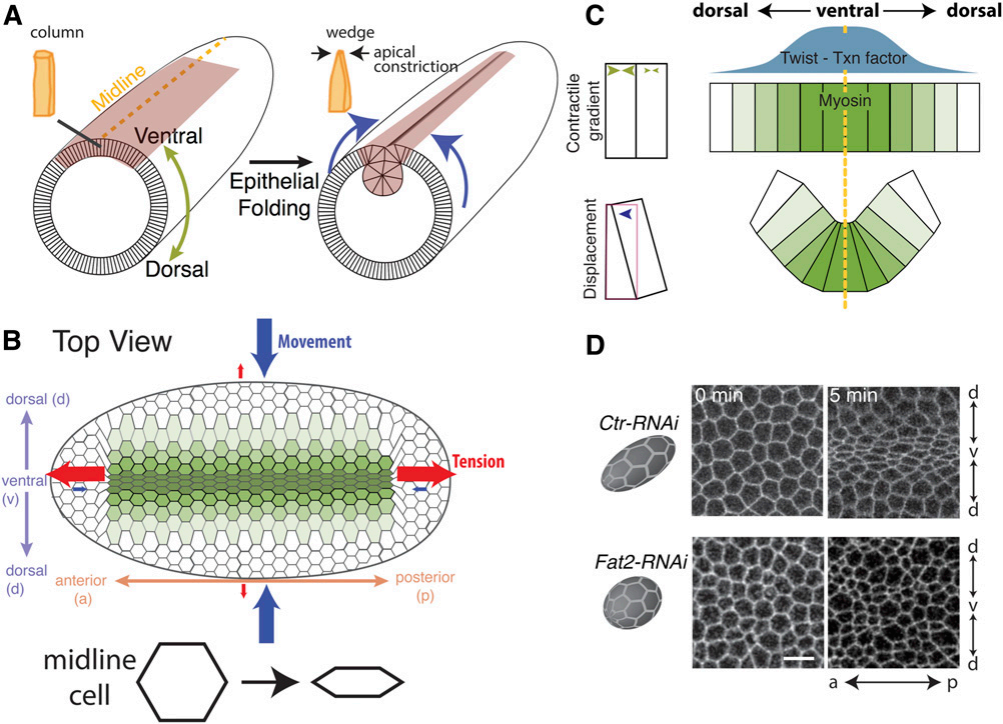
The mechanics of *Drosophila* mesoderm invagination. (A) Cartoon showing cell and tissue shape changes during mesoderm invagination. Orange cells illustrate cell shape. Blue arrows show movement of ectoderm tissue during mesoderm invagination (maroon). Note that ventral side is shown facing up to highlight the mesoderm. (B) Birds-eye view of presumptive mesoderm. Green illustrates the level of cell contractility. Red arrows denote tension and blue arrows denote movement. (C) Cross-section view of mesoderm cells during invagination. A gradient in *twist* expression (blue curve) results in a gradient in myosin 2 contractility (green), which promotes efficient apical constriction in the ventral–dorsal direction. (D) Apical constriction anisotropy depends on embryo shape. Images are subapical views of cells showing cell outlines. In the control, ellipsoidal embryos (Ctr-RNAi), cells constrict mostly in the ventral–dorsal direction. In round embryos (Fat2-RNAi), cell constrict isotropically. Images are reproduced from Chanet *et al.* (2017). Bar, 10 μm.

The result of integrating force across the tissue is the generation of global tissue movement and mechanical tension. These two outcomes tend to be anticorrelated: tension is highest when movement is restrained, and movement is often associated with lower tension. Mesoderm invagination is associated with anisotropic tension, with the highest tension oriented along the anterior-posterior axis, along which cells fail to constrict efficiently (Figure 6, A and B) (Martin *et al.* 2010). This anisotropic tension results from the shapes of the contractile domain and the embryo, which are longer along the anterior–posterior axis than the dorsal–ventral axis (Figure 6, A and B) (Spahn and Reuter 2013; Chanet *et al.* 2017; Guglielmi and De Renzis 2017). In addition, contractility is graded along the dorsal–ventral axis, which means that there is a force imbalance between cells tugging along that direction (Figure 6, B and C) (Spahn and Reuter 2013; Heer *et al.* 2017; Lim *et al.* 2017). The force balance along the anterior–posterior axis means that there is greater resistance to apical constriction along this axis, which results in higher tension and less cell shape change. Thus, the anisotropic tension in the mesoderm causes anisotropic apical constriction, with ventral midline cells remaining more elongated along the anterior–posterior axis, and more apically constricted and wedge-shaped along the dorsal–ventral axis (Figure 6, B and D, control) (Sweeton *et al.* 1991; Martin *et al.* 2010). This anisotropy in tension and cell shape depends on ellipsoidal embryo shape: changing the embryo to a more spherical shape disrupts this anisotropy, thereby illustrating the interdependence between cell and embryo form (Figure 6D) (Chanet *et al.* 2017). Thus, embryo and tissue shape feeds back on cell behavior, resulting in wedge-shaped rather than cone-shaped cells and a dorsal–ventral axis of curvature in the mesoderm (Figure 6, A and B).

### Mechanosensing during *Drosophila gastrulation*

**Figure 7:**
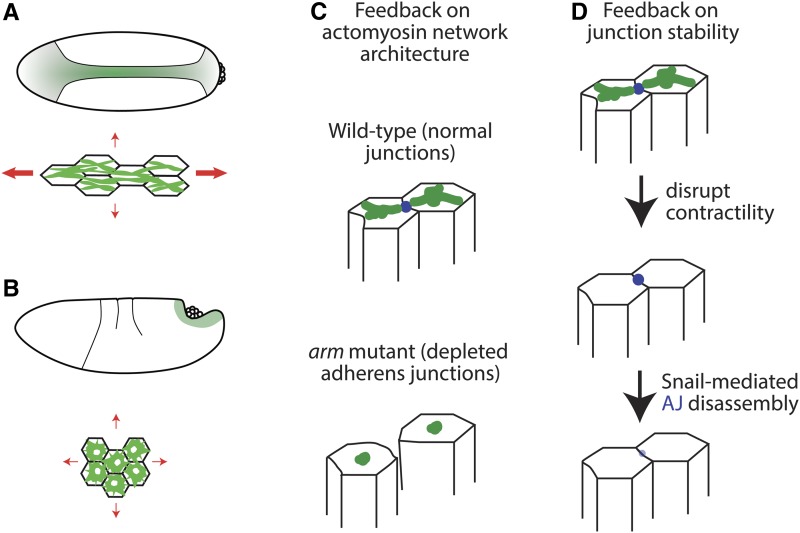
Mechanical feedback mechanisms in *Drosophila* gastrulation. (A) Cartoon illustrating the apical myosin 2 meshwork during mesoderm invagination. Note myosin 2 fibers oriented along the anterior–posterior axis are formed. Red arrows denote tension and, thus, resistance to contraction. (B) Cartoon illustrating the apical myosin 2 meshwork during posterior endoderm invagination. Note tension is isotropic (red arrows) and myosin 2 rings are formed. (C) Integration of mechanical force is necessary for apical myosin 2 meshwork structure. Top image illustrates a wild-type meshwork while bottom image illustrates the change in meshwork morphology in response to disrupting adherens junctions (*i.e.*, *armadillo* mutants). (D) Mechanical force is required for adherens junction stability. Cartoon illustrates the effect of disrupting contractility: adherens junctions disassemble in the absence of contractility.

In addition to generating force, the cytoskeleton and adherens junctions also respond to force through mechanosensing or mechanotransduction mechanisms (Hannezo and Heisenberg 2019). During gastrulation, the surrounding mechanical constraints to invagination regulate the geometrical properties of cells and their internal cytoarchitecture, suggesting long-range mechanical coupling and feedback (Chanet *et al.* 2017). As described above, the higher tension along the long axis of the embryo prevents cells from constricting along this axis, resulting in an elongated cell shape (Figure 7A) (Martin *et al.* 2010). In contrast, endoderm invagination is associated with low isotropic tension, leading to isotropic apical constriction (Figure 7B) (Chanet *et al.* 2017). In the mesoderm, myosin 2-containing fibers that connect between cells align and straighten with the axis of tension in the embryo (Figure 7A) (Chanet *et al.* 2017; Yevick *et al.* 2019). In contrast, perturbations that result in isotropic resistance to constriction or the natural process of endoderm invagination, result in the formation of myosin 2-containing rings (Figure 7B) (Chanet *et al.* 2017). Mechanical signals between cells have also been suggested to be responsible for inducing apical myosin 2 accumulation by inhibiting Fog/receptor endocytosis (Pouille *et al.* 2009; Mitrossilis *et al.* 2017). However, in the mesoderm, Fog activity is not required for invagination or myosin 2 stabilization, suggesting that mechanical feedback is not the primary mechanism of myosin 2 induction (Costa *et al.* 1994; Kolsch *et al.* 2007; Xie *et al.* 2016). Adherens junctions and the mechanical integration between cells are similarly not required for mesodermal apical myosin 2 accumulation, but do strongly impact actomyosin network geometry (Dawes-Hoang *et al.* 2005; Sawyer *et al.* 2009; Martin *et al.* 2010). In the absence of counterbalancing forces mediated by adherens junctions, myosin 2 fibers contract into medioapical spots (Figure 5B and Figure 7C). These data suggest that, in mesoderm cells, myosin 2 activation is cell autonomous, but that apical actomyosin network morphology and alignment depends strongly on mechanical context through force balance (Figure 7, A and B).

Mechanical feedback can also operate at the level of intercellular attachments. Recent work has shown that adherens junctions function as mechanical integrators of contractility in a tissue (Lecuit and Yap 2015). During mesoderm invagination, myosin 2 contractility stabilizes adherens junctions in the face of Snail-mediated disassembly (Figure 7D) (Weng and Wieschaus 2016). Indeed, acute pharmacological inhibition of ROCK decreases E-cadherin levels in spot junctions within 1 min of inhibition, demonstrating the dependence of adherens junction stability on myosin 2 contractility and a continuous pulling force (Coravos and Martin 2016).

## Tissue-Wide Mechanical Coupling of Morphogenetic Movements During Gastrulation

Like most other events in development, gastrulation does not occur in isolation. Presumptive mesoderm and endoderm tissues are connected to ectoderm tissue, and movements of mesoderm and endoderm depend on the deformability and other responses of the neighboring tissues. This coupling is illustrated by the fact that gastrulation is accompanied by global morphogenetic flows that occur across the entire embryo (Lye *et al.* 2015; Rauzi *et al.* 2015; Streichan *et al.* 2018). The global morphogenetic flows that occur in the embryo before and after mesoderm invagination are predicted with >>90% accuracy by the spatial distribution of myosin 2 across the embryo and its anisotropy (*i.e.*, orientation), suggesting that unbalanced myosin 2 activity promotes global tissue movement (Streichan *et al.* 2018).

One consequence of the invagination of mesoderm and endoderm tissues is that the remaining ectoderm must fill the significant surface void left by the internalization of these cells. Mesoderm invagination induces a dorsal–ventral tensile stretch on lateral germband cells (Lye *et al.* 2015). However, it appears that the cells that compensate most for mesoderm invagination are the dorsal-most cells, which stretch considerably in response to mesoderm invagination (Rauzi *et al.* 2015).

Posterior midgut invagination results in anterior–posterior tensile stress in the ectoderm (Collinet *et al.* 2015; Lye *et al.* 2015). Associated with the anterior–posterior elongation of ectoderm cells is the intercalation of these cells to elongate the germband, which allows the endoderm to internalize fully. Germband extension involves the planar polarized distribution of myosin 2, which causes junctions with one type of orientation to contract, while junctions with an orthogonal orientation elongate, resulting in directional cell intercalation and tissue elongation along the anterior–posterior axis (Irvine and Wieschaus 1994; Bertet *et al.* 2004; Zallen and Wieschaus 2004). *Drosophila* germband extension also involves planar polarized protrusions typical of cell crawling behavior, which could contribute to, or even lead, cell intercalation movements (Sun *et al.* 2017). Given that anterior–posterior cell stretching is intensified when cell intercalation is blocked, it is possible that germband elongation relieves stress in the ectoderm to allow the posterior midgut to efficiently internalize (Butler *et al.* 2009; Lye *et al.* 2015). Importantly, it has been shown that elongation of anterior–posterior oriented junctions, rather than shrinkage of myosin 2-containing dorsal–ventral oriented junctions, requires external pulling force from posterior midgut formation, suggesting that tissue elongation requires the combination of cell-scale forces that shrink dorsal–ventral oriented junctions and tissue-scale forces that promote elongation of anterior–posterior oriented junctions (Collinet *et al.* 2015).

## Consequences of Apical Constriction: Invagination *vs.* Ingression

Apical constriction of cells in an epithelial monolayer is associated with cell and tissue shape changes (*i.e.*, folding), as well as cell elimination or extrusion from the monolayer (Heer and Martin 2017; Fadul and Rosenblatt 2018). In contrast to the coordinated folding and invagination of the *Drosophila* mesoderm, the inward movement of individual cells or cell ingression is a feature of gastrulation in other animals (Lee *et al.* 2006; Wu and McClay 2007; Roh-Johnson *et al.* 2012; Williams *et al.* 2012). What determines whether cells completely constrict and are eliminated from the epithelium, or undergo coordinated, but incomplete, constriction and maintain tissue integrity? While the factors that determine the extent of apical constriction in different contexts are still unclear, several experiments have shed light on critical processes that promote tissue invagination.

Apical constriction initially leads to an apical–basal elongation of mesoderm cells (Sweeton *et al.* 1991; Gelbart *et al.* 2012). It has been suggested that constriction of the cell apex could be balanced by passive forces in the cell, such as hydrodynamic forces in the cytoplasm or by basal or lateral cortex tension. Consistent with the possibility that cytoplasmic pressure resists deformation and enables apical forces to be propagated basally, the volume of mesoderm cells remains more-or-less constant during apical constriction (Gelbart *et al.* 2012). Force from apical constriction induces hydrodynamic flows that lead to nuclear movement and apical–basal elongation (Gelbart *et al.* 2012; He *et al.* 2014). As mesoderm cells achieve a more wedge-shaped morphology they undergo basal expansion and apical–basal shortening, which is correlated with basal myosin 2 depletion (Polyakov *et al.* 2014). Indeed, ectopic myosin 2 activation after cell lengthening inhibits apical–basal cell shortening and tissue invagination, demonstrating a similarly important role for basal relaxation in cells achieving a final wedge shape and tissue invagination (Krueger *et al.* 2018).

Several mechanical models have been put forth to describe the process of mesoderm invagination, and these has been reviewed in detail by Rauzi and colleagues (Rauzi *et al.* 2013). A key feature of many of these models is that apical constriction shrinks the outer, apical surface relative to the inner, basal surface, which generates an inward curvature when the two surfaces are connected. In the simplest case, this principle can create curvature without accounting for other cell shape changes (Heer *et al.* 2017), although these other shape changes (*e.g.*, apical–basal forces and forces from ectoderm cells) are likely to affect the speed or the extent of invagination (Conte *et al.* 2012; Perez-Mockus *et al.* 2017; Gracia *et al.* 2019). Consistent with the proposed key role for apical constriction, the patterned optogenetic activation of RhoA in the early embryo can induce ectopic invaginations (Izquierdo *et al.* 2018). While these ectopic invaginations do not reproduce the exact shape of the invaginated mesoderm, these experiments nevertheless illustrate that apical myosin 2 activation plays a key role in initiating cell invagination.

**Figure 8:**
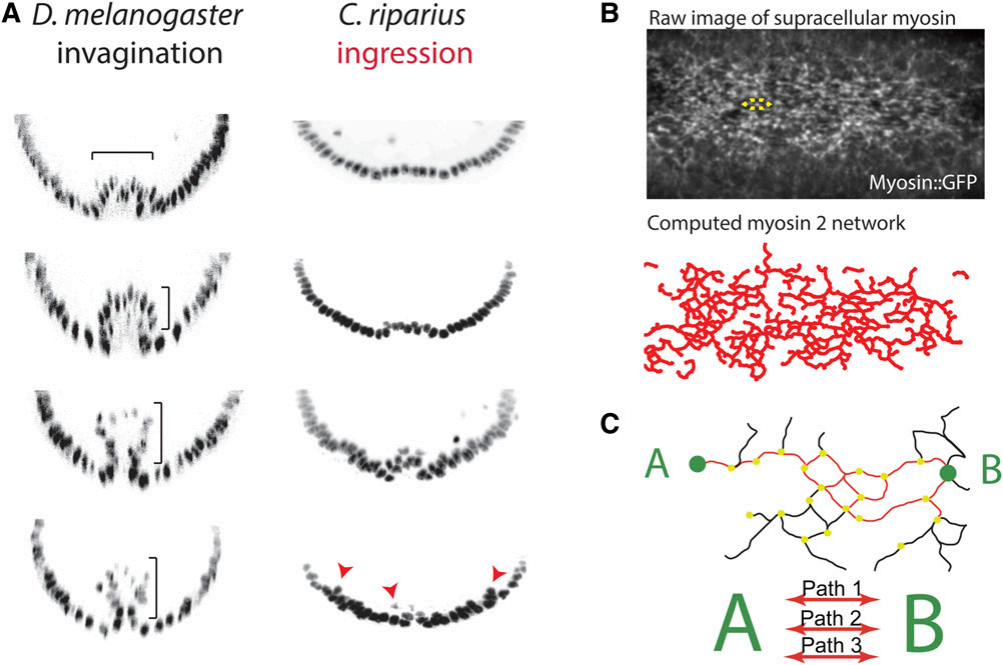
Mechanisms that promote collective cell invagination. (A) Cross-section images of embryos with labeled nuclei comparing *D. melanogaster* and *C. riparius* mesoderm internalization. Bracket shows collective tissue invagination of *Drosophila* and arrows show individual cell ingression in *C. riparius*. Images are reproduced from Urbansky *et al.* (2016). (B) Image is a Z-projection of sqh::GFP showing the medioapical supracellular myosin 2 meshwork. The approximate size of an individual cell is highlighted by the yellow dotted line. A computed trace of the myosin 2 network is below the raw image. Images are from one of the data sets used in Yevick *et al.* (2019). (C) Cartoon illustrating structural redundancy of the supracellular myosin 2 network. Reproduced part of Graphical abstract from Yevick *et al.* (2019).

Insight into why apical constriction leads to invagination, rather than ingression, has also come from comparing gastrulation in *Drosophila* to that in other insects such as the midge, *Chironomus riparius*. *C. riparius* and *D. melanogaster* last shared a common ancestor ∼250 MYA and exhibit different modes of mesoderm internalization (Urbansky *et al.* 2016). *D. melanogaster* exhibits epithelial folding and invagination as discussed in this chapter (Leptin and Grunewald 1990; Sweeton *et al.* 1991). In contrast, *C. riparius* exhibits cell ingression during its gastrulation (Figure 8A). The presumptive mesoderm of both *C. riparius* and *D. melanogaster* express *twist* and *snail*, and these transcription factors are also required for mesoderm internalization of *C. riparius* (Urbansky *et al.* 2016). The main difference in mesoderm gene expression between these two species is that *C. riparius* fails to express *fog* and *T48*, which together activate sustained actomyosin contractility in *Drosophila* mesoderm cells (Figure 2B) (Urbansky *et al.* 2016). Interestingly, ectopic expression (*i.e.*, not restricted to mesoderm) of either *fog* or *T48* in *C. riparius* changes mesoderm internalization from ingression to invagination mode (Urbansky *et al.* 2016).

In contrast to *C. riparius*, Fog signaling plays a role in mesoderm internalization for the flour beetle, *Tribolium castaneum*. In *T. castaneum*, the homologs of *twi*, *cta*, *fog*, and *mist* are expressed in the mesoderm, similar to *Drosophila* (Handel *et al.* 2005; Benton *et al.* 2019). Furthermore, *fog* depletion in *T. castaneum* delays mesoderm internalization and *T. castaneum* mesoderm cells undergo pulsatile contractions, similar to *Drosophila* (Benton *et al.* 2019). Interestingly, Fog signaling in *T. castaneum* and several other insect species plays a role in cellularization, suggesting that this might be the ancestral function of the Fog pathway (Benton *et al.* 2019).

Is there an advantage for mesoderm invagination occurring collectively *vs.* stochastically? Urbansky and colleagues noted that *Drosophila* mesoderm invagination occurs faster than *C. riparius* mesoderm ingression, and suggested that invagination might provide a more robust mechanism for internalization (Urbansky *et al.* 2016). Indeed, recent work suggests that the network of actomyosin connections that forms across the *Drosophila* presumptive mesoderm tissue promotes the robustness of the invagination process in several ways (Figure 8B) (Yevick *et al.* 2019). First, redundancy in mechanical connections between cells ensures overall network connectivity and tissue function in the face of local or cell damage (Figure 8C). Second, directional connectivity and stiffening of the network along the anterior–posterior axis enables furrow formation at lower contractility levels and ensures proper furrow/fold orientation.

In addition to the expression of *fog* and *T48*, other mechanisms exist to prevent cell extrusion during invagination. During normal *Drosophila* mesoderm invagination, tissue integrity is maintained during folding, and is only lost after internalization when cells undergo epithelial-to-mesenchymal transition (EMT) (Figure 1B) (McMahon *et al.* 2008, 2010; Clark *et al.* 2011). Depletion of the Abelson nonreceptor tyrosine kinase (Abl) results in abnormal extrusion of mesoderm cells as they invaginate (Jodoin and Martin 2016). Abl regulates numerous cellular processes and components, including the cytoskeleton, where Abl negatively regulates an actin assembly factor, Enabled (Ena) (Gertler *et al.* 1990, 1995). In *Drosophila* blastoderm cells, Abl loss results in excessive apical microvilli at the expense of other actin structures (Grevengoed *et al.* 2003). Furthermore, Abl loss in mesoderm cells results in ectopic later F-actin (Fox and Peifer 2007). Cell extrusion and other mesoderm defects result from abnormal Ena activity because these phenotypes are suppressed by codepletion of Ena (Fox and Peifer 2007; Jodoin and Martin 2016). Cell extrusion that results from Abl depletion is accompanied by the disruption of apical–basal cell polarity, suggesting that cells undergo a premature EMT-like process (Jodoin and Martin 2016). Therefore, in *Drosophila*, it appears that mechanisms are in place to properly time EMT, and, thus, to maintain tissue integrity during mesoderm invagination.
